# Saccular Transcriptome Profiles of the Seasonal Breeding Plainfin Midshipman Fish (*Porichthys notatus*), a Teleost with Divergent Sexual Phenotypes

**DOI:** 10.1371/journal.pone.0142814

**Published:** 2015-11-11

**Authors:** Joshua Faber-Hammond, Manoj P. Samanta, Elizabeth A. Whitchurch, Dustin Manning, Joseph A. Sisneros, Allison B. Coffin

**Affiliations:** 1 College of Arts and Sciences, Washington State University, Vancouver, WA, United States of America; 2 Systemix Institute, Redmond, WA, United States of America; 3 Department of Biological Sciences, Humboldt State University, Arcata, CA, United States of America; 4 Department of Psychology, University of Washington, Seattle, WA, United States of America; 5 Department of Integrative Physiology and Neuroscience, Washington State University, Vancouver, WA, United States of America; Virginia Commonwealth University, UNITED STATES

## Abstract

Acoustic communication is essential for the reproductive success of the plainfin midshipman fish (*Porichthys notatus*). During the breeding season, type I males use acoustic cues to advertise nest location to potential mates, creating an audible signal that attracts reproductive females. Type II (sneaker) males also likely use this social acoustic signal to find breeding pairs from which to steal fertilizations. Estrogen-induced changes in the auditory system of breeding females are thought to enhance neural encoding of the advertisement call, and recent anatomical data suggest the saccule (the main auditory end organ) as one possible target for this seasonal modulation. Here we describe saccular transcriptomes from all three sexual phenotypes (females, type I and II males) collected during the breeding season as a first step in understanding the mechanisms underlying sexual phenotype-specific and seasonal differences in auditory function. We used RNA-Seq on the Ion Torrent platform to create a combined transcriptome dataset containing over 79,000 assembled transcripts representing almost 9,000 unique annotated genes. These identified genes include several with known inner ear function and multiple steroid hormone receptors. Transcripts most closely matched to published genomes of nile tilapia and large yellow croaker, inconsistent with the phylogenetic relationship between these species but consistent with the importance of acoustic communication in their life-history strategies. We then compared the RNA-Seq results from the saccules of reproductive females with a separate transcriptome from the non-reproductive female phenotype and found over 700 differentially expressed transcripts, including members of the Wnt and Notch signaling pathways that mediate cell proliferation and hair cell addition in the inner ear. These data constitute a valuable resource for furthering our understanding of the molecular basis for peripheral auditory function as well as a range of future midshipman and cross-species comparative studies of the auditory periphery.

## Introduction

The plainfin midshipman fish (*Porichthys notatus*) is an excellent model organism for studies of vocal-acoustic communication, in part because acoustic communication is essential to their social and reproductive behaviors [1,2]. Type I (“parental”) males nest in the rocky intertidal zone during the spring and summer breeding season, where they produce advertisement calls to attract females. This mate call is produced via rapid contraction of the sonic muscles associated with the swim bladder, resulting in a characteristic “hum” with a fundamental frequency that ranges from 80–100 Hz depending on water temperature [3]. While the fundamental frequency is quickly attenuated, the higher harmonic components of the hum propagate well in the shallow-water breeding environment. As these fish are nocturnal spawners, vocal-acoustic signals are likely the primary sensory cue used by gravid females to detect and locate mates.

Female midshipman show a robust behavioral response to both natural and synthetic hums, and physiological responses to the hum are encoded by the saccule, which is the primary auditory organ in this and most other fish species [4–7]. Interestingly, the female’s auditory system demonstrates adaptive seasonal plasticity that includes enhanced encoding of the hum’s higher frequency harmonics and a saccular-specific increase in sensory hair cell density during the breeding season [8–11]. Recent work demonstrates similar seasonal physiological plasticity of the auditory system in type I males and in other vertebrate taxa including birds and amphibians, suggesting that seasonal auditory plasticity may be a general vertebrate trait [12–14].

In addition to the type I males, a subset of male midshipman fish use alternative mating tactics to achieve reproductive success. These small type II (“sneaker”) males, which superficially resemble females, steal fertilizations during spawning events between type I males and females. Type II males do not produce courtship vocalizations but instead invest more energy in gamete production [15,16].

In this study we used RNA-Seq to profile the saccular transcriptome from reproductive midshipman fish of all three sexual phenotypes: females, type I, and type II males. We also included saccular RNA-Seq data from the non-reproductive female phenotype as a first step in our seasonal comparison. Transcriptome-level analysis has been previously conducted in the inner ears of several vertebrates including mice, frogs (*Xenopus*), and zebrafish (*Danio rerio*) [17–22]. These studies were all conducted in species for which genomic sequences were available and examined an array of questions surrounding inner ear development, altered transcription in mutant lines, and inner ear responses to toxins [19, 21, 22]. Here we apply transcriptome profiling to investigate new questions related to the genetic mechanisms underlying sexual phenotypic differences in auditory function in a vocal teleost fish, for which no genome sequence is available. We employed next generation sequencing, allowing us to analyze the inner ear transcriptome for the plainfin midshipman. RNA-Seq has been successfully used in other non-traditional model organisms lacking sequenced genomes including the guppy (*Poecilia reticulata*), and recently, in an innovative study of vocal motor areas in the plainfin midshipman hindbrain [23,24].

We obtained over 79,000 assembled transcripts (ATs) in our *de novo* construction of the midshipman saccular transcriptome, representing approximately 9,000 unique genes. This work sets the stage for future studies on cross-seasonal comparisons and for understanding the molecular events necessary for auditory plasticity to occur in this species, and perhaps more generally in vertebrates. In addition, the novel transcripts represent an untapped resource to study new inner ear genes.

## Materials and Methods

### Fish Collection and RNA extraction

Reproductive plainfin midshipman fish were collected in the summer of 2012 near Brinnon, WA by hand at low tide, when they reside in shallow pools under rocky nests. Animals were transported live back to the lab were they were euthanized in a water bath with 0.05% benzocaine (Sigma-Aldrich). Fish remained in benzocaine for 10 minutes after cessation of opercular movement. Fish were then weighed, measured, and the saccules quickly dissected from the head. Saccules were stored in RNALater (Sigma-Aldrich) at -20°C for up to one week prior to microdissection of the saccular epithelium (while in RNA Later) and RNA extraction. All animal procedures were approved by the University of Washington Institutional Animal Care and Use Committee (permit 4079–01) or Washington State University Institutional Animal Care and Use Committee (permit 04434–003). Fish collecting was conducted under field permit 12–192 granted by the Washington Department of Fish and Wildlife. This permit applies to both hand collection from nests and otter trawls.

For RNA extraction we combined five saccules from three reproductive type I males (standard length (SL) 14.2–17.5 cm), seven saccules from four reproductive type II males (SL 11.7–12.8 cm), and six saccules from three reproductive females (SL 13.1–16.9 cm). As a proxy for relative reproductive state we calculated the gonadosomatic index (GSI) for each fish, defined here as 100 * gonad mass/(body mass-gonad mass) [25]. GSI range for each reproductive sexual phenotype was as follows: type I males 0.85–2.53, type II males 10.03–13.70, and females 3.07–24.89. Although one reproductive female had a low GSI value, we consider her to be in reproductive condition (but “spent” or devoid of gravid eggs), because she was removed from a type I male’s nest and had likely spawned just prior to capture.

Micro-dissected saccules were combined as described above, then crushed with a pestle to lyse the tissue. mRNA was purified from the lysate using the GeneElute mini-prep mRNA kit (Sigma-Aldrich) according to the manufacturer’s protocol.

### Sequencing

Saccular RNA samples from all three sexual phenotypes (type I male, type II male, reproductive female) were sent to the Laboratory of Biotechnology and Bioanalysis at WSU Pullman for Ion Torrent sequencing. One nanogram of mRNA was used to construct RNA-seq libraries using the Ion Total RNA Seq V2 kit (Life Technologies) low input protocol, with the exception that AMPureXP SPRI beads (Beckmann Coulter) were used for all purifications during library construction. Final library size selection was achieved with 0.7X AMpureXP. The libraries were quantified by qPCR and sequenced separately on an Ion Torrent PGM using a single Ion 318 chip for each sample and sequencing beads produced on an Ion OneTouchDL using 200bp chemistry.

### Transcriptome Assembly

Ion Torrent data were assembled using MIRA (version 3.4.0.1). A majority of ribosomal RNA (rRNA) sequences were removed from raw sequence datasets using the short sequence alignment algorithm Bowtie2 (version 2.0.2) prior to assembly in MIRA. A Bowtie2 index was created for filtering rRNA/rDNA sequences by searching the GenBank NR (non-redundant) database for “fish rDNA”, downloading the resulting 2,377 FASTA sequences, then running the bowtie2-build command. Each one of these sequences was checked to ensure the GenBank search did not yield any non-ribosomal sequences. Bowtie2 was run to align each Ion Torrent dataset to the “fish rDNA” index using the—*fast-local* parameter preset. Then, all sequences *without* homology to the “fish rDNA” index were exported for transcriptome assembly.

The—*fast-local* preset was chosen to ensure that Bowtie2 only found very strong alignments because it was more desirable to leave a portion of rRNA sequences in the raw datasets than to remove non*-*ribosomal sequences inadvertently. To examine the efficacy of the removal of ribosomal sequences, BLAST2GO pro (version 2.6.4) was used to run BLASTN analysis on a subset of 497 reads removed from the type I male dataset, as these presumably represent ribosomal RNA. Only 2/497 (0.402%) reads did not show homology to known ribosomal sequence in the NR database, nor did they show homology to any known sequence. Upon examination, these two reads both contained long stretches of repeats. This analysis lends confidence to our method of removing rRNA from the transcriptome assembly.

Each Ion Torrent dataset (type I male, type II male, female) with rRNA sequences removed was assembled with MIRA using the following job parameters: *denovo*, *est*, *accurate*, *iontor*. “Denovo” assembles the transcriptome in the absence of prior scaffold information. “EST” assembles the data as expressed sequence tags (rather than as genomic DNA), “accurate” is the MIRA default for complete dataset assembly, and “iontor” specifies the sequencing technology used to generate the dataset. In this assembly, all sequences less than 40 bp were ignored, and a minimum of two transcripts had to align in order for MIRA to include the contig in the assembly. Next, the three “EST” assemblies were used together as inputs for a combined MIRA assembly using the same parameters. This significantly increased average sequence length and decreased the total number of assembled transcripts.

Bowtie2-build was used to create indexes for the separate female, type I male, and type II male sequence assemblies. Those indexes were then used to extract matching sequences from the combined reference assembly using Bowtie2 (—*very-sensitive-local* parameters for increased search strength). The resulting three datasets allowed for between-dataset comparisons and were used for downstream “sexual phenotype” analyses. As an additional quality control for the MIRA assembly, single-end libraries were assembled together using Trinity (trinityrnaseq_r20140717, default parameters), which generated 157,000 assembled sequences excluding isoforms and 207,351 sequences including isoforms. Given the concordance between assemblies we have elected to use the MIRA assembly for functional annotation. Data are available at NCBI under the Bioproject accession PRJNA200442.

### Gene Annotation

General statistics for sequence datasets were generated using PrinSeq (version 0.20). These statistics include mean, N50, minimum, maximum, and range for both sequence length and GC content.

BLAST2GO pro was used for BLASTX analysis, mapping, and annotation of the combined reference assembled transcript (AT) dataset. For those ATs that did not yield GO-terms (gene ontology terms) following this BLAST2GO pipeline, the InterProScan database was checked for possible homologies and annotations. Those ATs that showed no protein homology in either analysis were subjected to BLASTN analysis. BLASTN results helped to determine the identity of additional ATs, but annotations did not provide GO terms for functional analysis. As there were many instances where multiple ATs showed homology to a single gene, the list was collapsed so that genes were only counted once in subsequent functional analysis.

Functional categories of interest were developed for grouping ATs from the combined and sexual phenotype-specific datasets by key-words within GO-terms (Table 1). Because a single AT often yields multiple GO-terms, the functional categories are not mutually exclusive. Searches were conducted using the “contains” and “does not contain” filters in Microsoft Excel. For each search filter the results were visually inspected to ensure the lists did not contain unintended GO-terms (*e*.*g*., contains “ear” and does not contain “nuclear”).

**Table 1:** Search classification terms for functional AT categories of the combined midshipman saccular transcriptome assembly.

Category	Search terms
**Signal transduction**	Signal transduction, signal transducer, tyrosine phosphatase, tyrosine kinase, signaling pathway, growth factor, cell communication, G-protein coupled receptor, serine/threonine kinase
**Actin-associated**	Actin, myosin
**Ion channels/transporters**	Ion channel, ion transport, channel, ion exchanger, voltage-gated, ligand-gated, symporter, antiporter, sodium, potassium, chloride, anion, cation
**Calcium regulation**	Calcium, calcium ion binding
**Cell death**	Apoptotic, death, bcl, p53, calpain, cathepsin, fas, apoptosis
**Cell proliferation**	Cell proliferation, cell cycle, cyclin, mitotic, mitosis, cell division, cytokinesis, G2/M, cohesin, cdc, cdk
**Inner ear**	Ear, hearing, sound, auditory, semicircular canal, otolith, tectorin, otogelin, otoferlin, usherin, usher, espin, oto, cochlin, otic
**Neuron**	Neuron, neural, axon, dendrite, synapse, synaptic, nerve, neurotransmitter, nervous system, elav, dopamine, serotonin, glutamate, GABA, glycine, ganglion, myelin, acetylcholine, spine dendritic, neuro, synap,
**Hormone associated**	Estrogen, androgen, testosterone, thyroid, hormone, steroid, progest, estradiol, corticoid
**Transcription factors**	Transcription factor, sequence-specific, DNA-dependent, transcription, homeobox, hox, sox, forkhead, x-box, e-box

A list of 109 known inner ear and/or deafness-related genes was compiled from the Hereditary Hearing Loss (HHL) database (http://hereditaryhearingloss.org; accessed 11/2/2012) to query the AT sets for genes linked to inner ear function. Gene names from the HHL database were used to search GenBank for mRNA sequences from other vertebrates, as none of these genes had been previously sequenced in midshipman. Fish sequences were selected when possible. When GenBank searches yielded alternative splice variants or paralogs of a certain gene, all variants of that gene were downloaded for analysis. These sequences were used to create a FASTA file of ear-related genes that we could use to query our midshipman AT set. BLAST databases were generated for the combined and individual midshipman assemblies using the makeblastdb tool from the blast+ package (NCBI). The HHL-derived FASTA dataset was used to query the combined and individual midshipman datasets using stand-alone BLASTN searches. To determine the optimal BLASTN search parameters for queries across taxa, the word size and minimum BLAST scores were adjusted in a stepwise manner to maximize real hits while minimizing erroneous hits. This iterative process led us to set word size to 11 and BLAST results were filtered for only hits with a minimum score of 70.

To better understand the nature of both annotated and unknown transcripts within our dataset, optimal BLASTN parameters from the prior analysis were used to query all ATs against nine sequenced teleost genomes: zebrafish (*Danio rerio)*, medaka (*Oryzias latipes)*, Atlantic salmon (*Salmo salar)*, Fugu (*Takifugu rubripes)*, spotted green pufferfish *(Tetraodon nigroviridis)*, large yellow croaker *(Larimichthys crocea)*, Nile tilapia (*Oreochromis niloticus)* platy (*Xiphophorus maculatus*) and three-spined stickleback (*Gasterosteus aculeatus*). This analysis leant additional support to our assembly and functional annotations.

### Expression analysis

For the expression analysis we compared gene expression between sexual phenotypes, and we included a fourth transcriptome dataset generated from non-reproductive female midshipman. Non-reproductive females were collected in December 2012 by otter trawl (R/V Kittiwake; Bio-Marine Enterprises) in Puget Sound near Edmonds, Washington at depths of 60–100 m. RNA extraction, sequencing, and assembly are as described above for the summer samples.

Kallisto (http://pachterlab.github.io/kallisto/) was used to compute expression levels in each of the four samples (type I, type II, reproductive female, non-reproductive female). First, an index was built using the *'kallisto index'* command with default k-mer size based on the transcripts assembled by MIRA. Next, *'kallisto quant'* was used to compute TPM (transcripts per million reads) expression levels of genes for each of the four samples. Additionally, we used the raw counts from Kallisto to determine significantly expressed transcripts using DESeq2 with the default analysis parameters and a significance level of p < 0.1 [26]. Functional information for differentially expressed genes was obtained from UniProt.

### RT-PCR validation

A subset of functionally significant ATs (myosin VIA, NADH dehydrogenase, estradiol 17β-dehydrogenase 12b, S100-A1-like, SPARC-like isoform 3, otogelin-like, otolin1, androgen receptor α, slit homolog 3) were selected for validation using RT-PCR. mRNA (extracted as described above) was reverse transcribed using GoScript Reverse Transcription System and oligo (dT) primers according to the standard manufacturer’s protocol (Promega, Madison, WI, USA). Amplification of cDNA was performed using GoTaq G2 Hot Start polymerase (Promega) with the following thermocycler parameters: 95°C for 2 min, followed by 35 cycles of: 95°C for 30 sec, 55°C–60°C (depending on primer pair; see Table 2) for 30 sec, and 72°C for 1 min. PCR products were gel extracted (QIAquick Gel Extraction Kit, Qiagen, Valencia, CA, USA), sequenced, and their identity verified by BLAST analysis.

**Table 2:** Primer sequences and annealing temperatures used for PCR reactions to amplify selected genes of interest. F: forward primer, R: reverse primer.

Gene of Interest	Annealing temp.	Primer Sequence
**Myosin VIa**	55°C	**F:** TCAAAGTCGAACAGGCGAAC
		**R:** CCAAGCGATGTCCAGAACCC
**Estradiol 17β-Dehydrogenase 12b**	60°C	**F:** CTCCAAGGCGTTTGTGGACT
		**R:** GGACGAAACCCATCCCCATC
**NADH Dehydrogenase**	55°C	**F:** TTCAACTCTCGTATTCGCATC
		**R:** GCCACCACACGCTTCAC
**S100-A1-like**	55°C	**F:** AAGGGGACAAGTACACGCTG
		**R:** TTCTGCGAGTCCACAATCCC
**SPARC-like isoform 3**	55°C	**F:** GGAGGAGACAGATGCTGAGG
		**R:** TTTGCCTTTCTTGCAGAGGT
**Otogelin-like**	55°C	**F:** TGCATCGACGTCATAGCTCC
		**R:** GTGACCTCCGTGGTTACCTG
**Otolin-1**	55°C	**F:** CGCCTACTCTCTGTCGCCTA
		**R:** GGACAGCACTCGCAAAAGTT
**Androgen Receptor-α**	55°C	**F:** CAGTGGAGGGCCTGAAGAAC
		**R:** TAGTCCAGCAGCTGTTGATG
**Slit homolog 3**	55°C	**F:** CCAGAATCACCAAGGTGGAC
		**R:** AAGCTCTGGCAGAAACTGGA

## Results

### Saccular transcriptome assembly

Unassembled Ion-Torrent datasets from reproductive type I male, type II male, and female saccules contained an average of 4,496,247.33 sequence reads with a mean read length of 193.87 bp (Table 3). After removing sequences with rRNA homology, the filtered datasets contained an average of 2,696,180.33 reads (~40% removed). The type I male assembly yielded 503,993 contigs with an average AT length of 240.69 bp. The type II male assembly contained 115,977 contigs with an average AT length of 218.11 bp. The female assembly consisted of 165,683 contigs with an average AT length of 201.37 bp. After combining the sexual phenotype-specific datasets for a secondary assembly, average contig length increased by 180.14 bp (relative to unassembled datasets) to 362.24 bp and had a mean GC content of 48.95%. The final combined assembly contained 79,814 ATs.

**Table 3:** Assembly statistics for the midshipman fish saccular transcriptome. Data are described for the raw RNA datasets, rRNA filtered datasets, sexual phenotype-specific assemblies, and the combined assembly.

	Mean length	Min length	Max length	N50 contig size	Mean GC content	Total # sequences	Total # bases
**Raw type I male**	201.19 ± 56.72 bp	18 bp	3008 bp	223 bp	52.38 ± 5.87%	4,295,038	864,138,918
**Raw type I male rRNA filtered**	200.49 ± 57.26 bp	18 bp	1024 bp	208 bp	52.14 ± 5.94%	3,984,080	798,778,434
**Raw type II male**	200.79 ± 67.02 bp	18 bp	3018 bp	209 bp	55.79 ± 4.75%	5,068,464	1,017,705,360
**Raw type II male rRNA filtered**	179.01 ± 79.41 bp	18 bp	3018 bp	208 bp	56.11 ± 6.19%	1,889,485	338,233,496
**Raw female**	179.63 ± 62.05 bp	18 bp	2519 bp	191 bp	55.51 ± 5.18%	4,125,240	741,028,157
**Raw female with rRNA filtered**	166.81 ± 66.65 bp	18 bp	1019 bp	181 bp	55.30 ± 6.19%	2,214,976	369,489,889
**Original type I male assembly**	240.69 ± 113.14 bp	40 bp	2364 bp	251 bp	47.13 ± 7.12%	503,993	121,303,896
**Original type II male assembly**	218.11 ± 101.08 bp	40 bp	3093 bp	272 bp	50.60 ± 8.05%	115,977	25,295,418
**Original female assembly**	201.37 ± 104.80 bp	39 bp	3273 bp	235 bp	48.99 ± 8.10%	165,683	33,363,065
**Combined assembly**	362.24 ± 167.41 bp	42 bp	3558 bp	399 bp	48.59 ± 6.75%	79,814	28.912,155

### Functional annotation

We annotated the 79,814 ATs using the BLAST2GO pipeline. 34,804 (43.6%) ATs yielded BLASTX hits to the GenBank NR database, and of these hits 14,241 (40.9%) represented unique gene names. A small subset of these 14,241 unique transcripts were likely represented more than once, as some gene names were slight variations on one another (*e*.*g*., myosin VIIa vs. myosin VIIa-isoform-partial). Therefore, the 14,241 figure represents an over-estimate of the number of unique transcripts annotated in the combined dataset. 11,221 of these unique genes had associated GO-terms for functional analysis.

**Fig 1:**
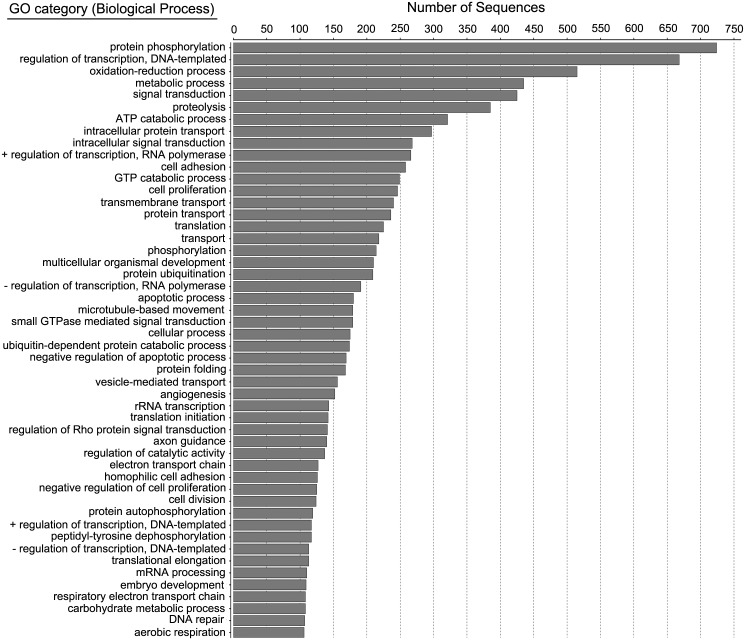
Gene Ontology (GO) term classification by biological process (BP) for the combined midshipman saccular transcriptome.

**Fig 2:**
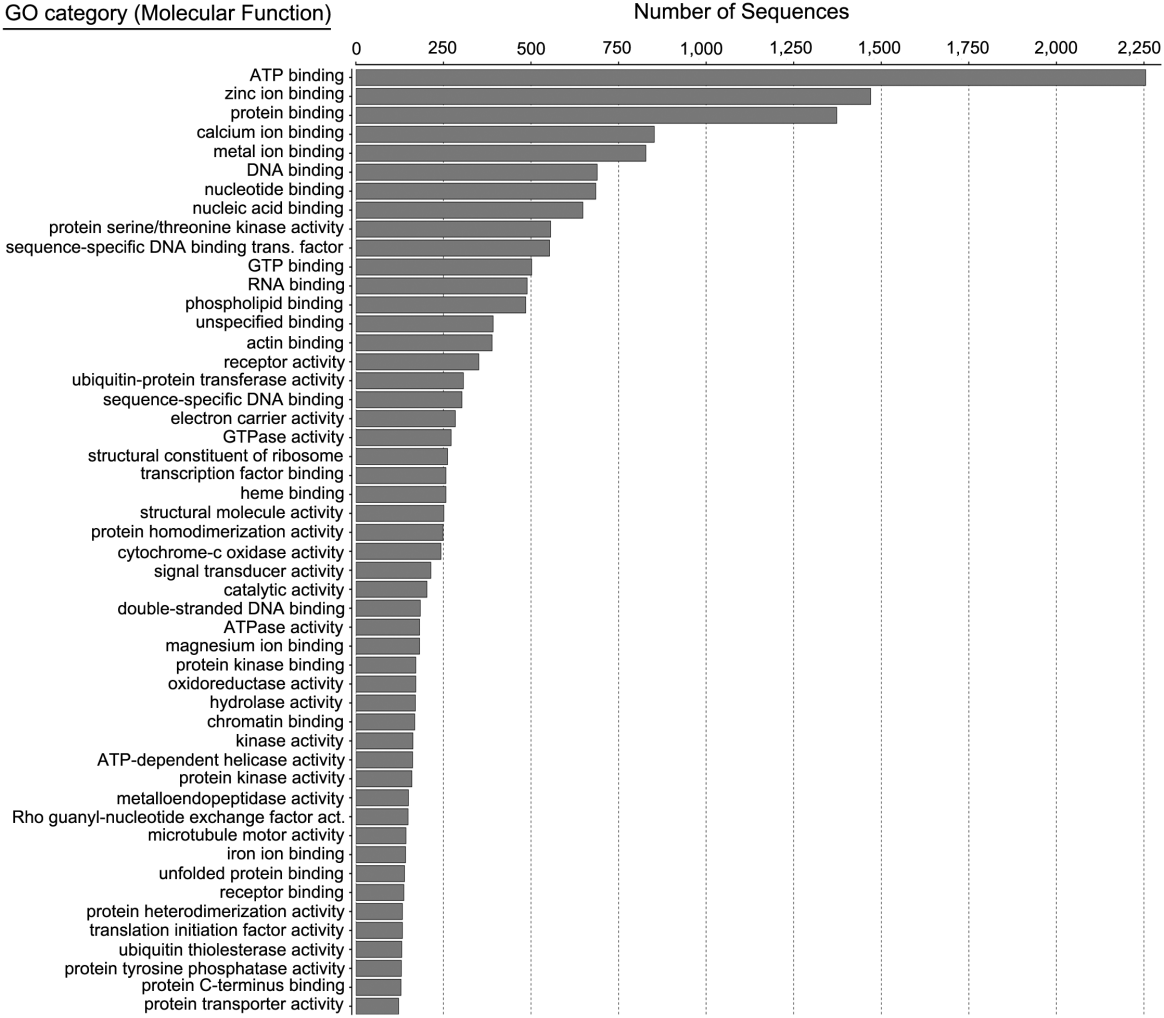
Gene Ontology (GO) term classification by molecular function (MF) for the combined midshipman saccular transcriptome.

Functional analysis was conducted for all transcripts that yielded GO-terms. Fig 1 shows the GO-term classification by biological process (BP) for the combined dataset, while Fig 2 shows the same classification by molecular function (MF). For biological process, the top GO-term categories were protein phosphorylation and DNA-binding transcriptional regulators, consistent with high levels of gene expression regulation and active cell signaling events. For molecular function, the bulk of ATs were classified as binding ATP, zinc, or calcium, again consistent with cell signaling regulation.

**Fig 3:**
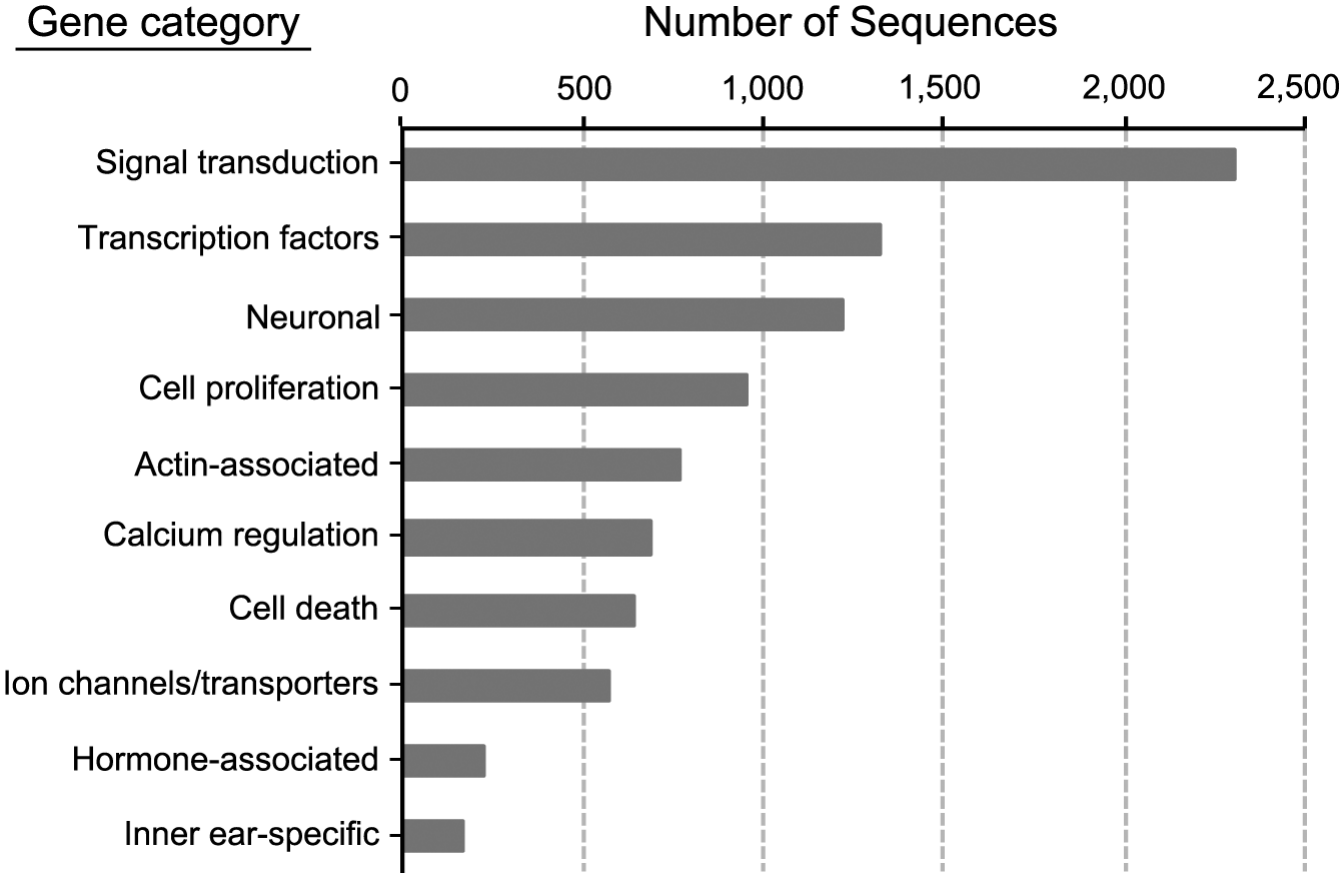
Functional analysis for all transcripts yielding gene ontology (GO) terms. The individual functional categories are not mutually exclusive, as many annotated genes have multiple gene ontologies (see Methods). ATs were classified in the narrowest appropriate category. Search terms for functional categories are located in Table 1.

We then composed categories that included multiple GO-terms in combinations that encompassed suites of genes with related functions, such as cell death, cell proliferation, or neuronal associations (see Table 1). These categories were selected to allow gross classification of groups of genes relevant to the inner ear. A high proportion of the annotated transcripts could be classified using our assigned categories (Fig 3). 5,327 genes fell into at least one functional category, which is 46.13% of all annotated genes with associated GO-terms. The manual annotation revealed 770 distinct ATs for actin-associated proteins, which are important because the actin-rich sensory hair bundle plays a critical role in transducing sound into neural responses [27]. 168 ATs had known inner ear function, including the transcription factor Brn3c (Pou4f3) and the molecular motor myosin VI, both of which cause deafness when mutated [28–30]. Another 228 ATs encode components of hormone signaling, including androgen and estrogen receptors and genes for steroid hormone metabolism. These latter ATs are of particular interest, as hormonal regulation of the auditory system has been previously reported for midshipman fish [9].

We further examined expression of known inner ear genes in our combined dataset to assess transcriptome quality. BLASTN and BLASTX analysis of deafness-related genes within the midshipman saccular transcriptome revealed that 73 of 109 sequences were found in our assembly (S1 Table). These deafness-related genes fall into several GO categories, including transcriptional regulation and otolith (ear stone) mineralization. The prevalence of deafness genes in our combined transcriptome, particularly in light of not having genomic midshipman sequence, offers confidence that this dataset likely contains the majority of all possible saccular transcripts in this species.

### Taxonomic comparison

**Fig 4:**
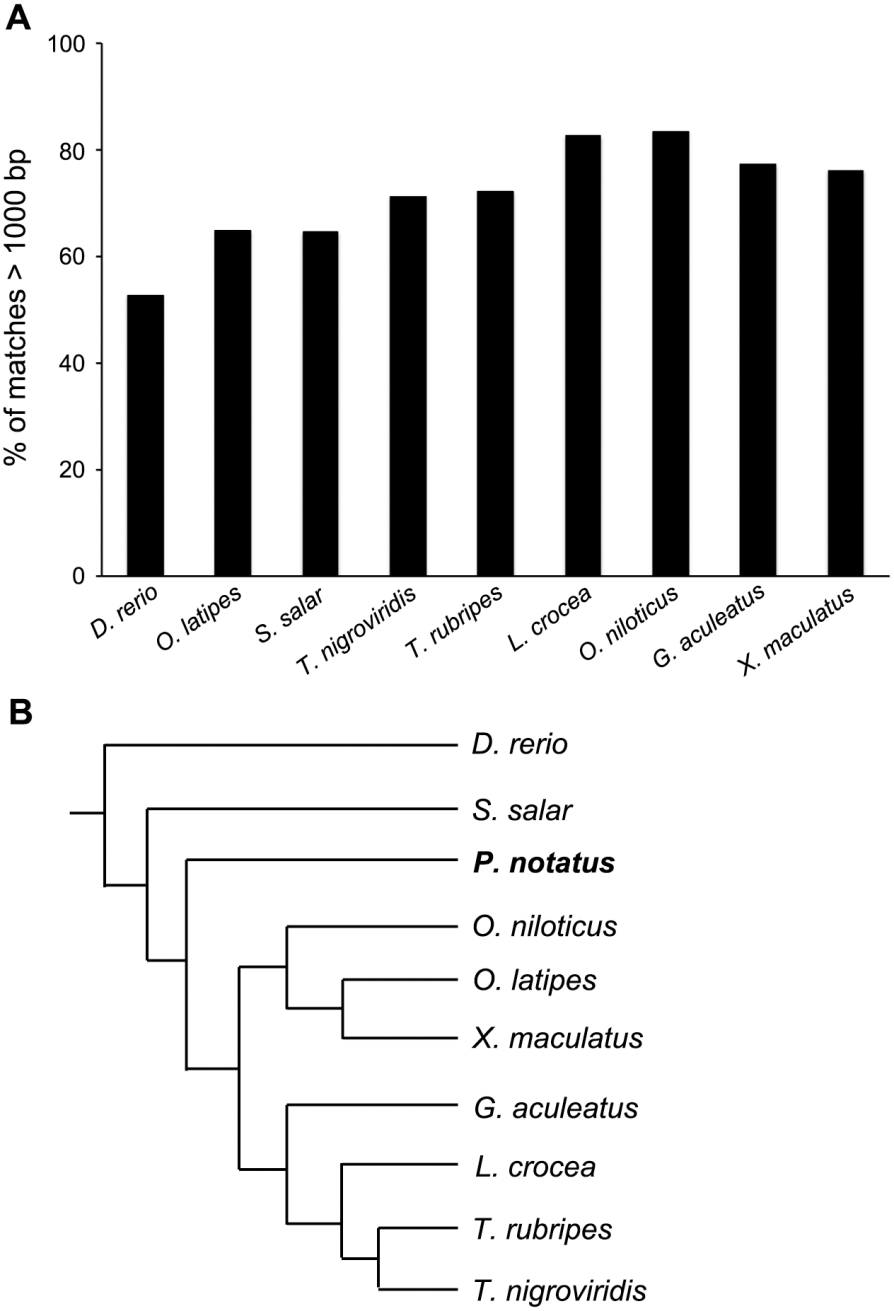
Comparison of *P*. *notatus* inner ear transcripts with other fish genomes. A) Analysis of the 460 midshipman sequences longer than 1000 bp (BLASTN with a cutoff of 1e-10). Midshipman transcripts most often match *L*. *crocea* (large yellow croaker) and *O*. *niloticus* (Nile tilapia) genomes. B) Phylogenetic relationships between *P*. *notatus* and the nine species used for genomic comparison. Phylogeny is based on [31]. Lines are not drawn to scale.

As there is no published genome for any batrachoidid fish, we compared our unknown ATs to nine published genomes from other fish taxa. We used only ATs >1000 bp for this analysis, as these likely represent full-length transcripts, although analysis with AT >500 bp yielded similar results (data not shown). Over 80% of these long ATs matched to sequences in either the *L*. *crocea* or *O*. *niloticus* genomes, while only 52.6% of ATs aligned to the *D*. *rerio* genome (BLASTN cutoff 1e-10, Fig 4). The phylogenetic relationships between *P*. *notatus* and the other nine species are shown in Fig 4B.

Due to the high degree of concordance between the *P*. *notatus* and *L*. *crocea* genomes, we further compared all assembled transcripts from *P*. *notatus* with the annotated genes of *L*. *crocea* using BLAST, rather than restricting our analysis to only transcripts larger than 1000 bp. Among the 79,814 ATs, 28,618 matched to 8,946 *L*. *crocea* genes by considering only the best hit for each AT (BLASTN cutoff 1e-10). In many cases, multiple *P*. *notatus* transcripts matched the same *L*. *crocea* gene, likely due to the fragmented nature of the *P*. *notatus* transcripts. This analysis gives us further confidence in our assembly and annotation.

### RT-PCR validation of saccular gene expression

**Fig 5:**
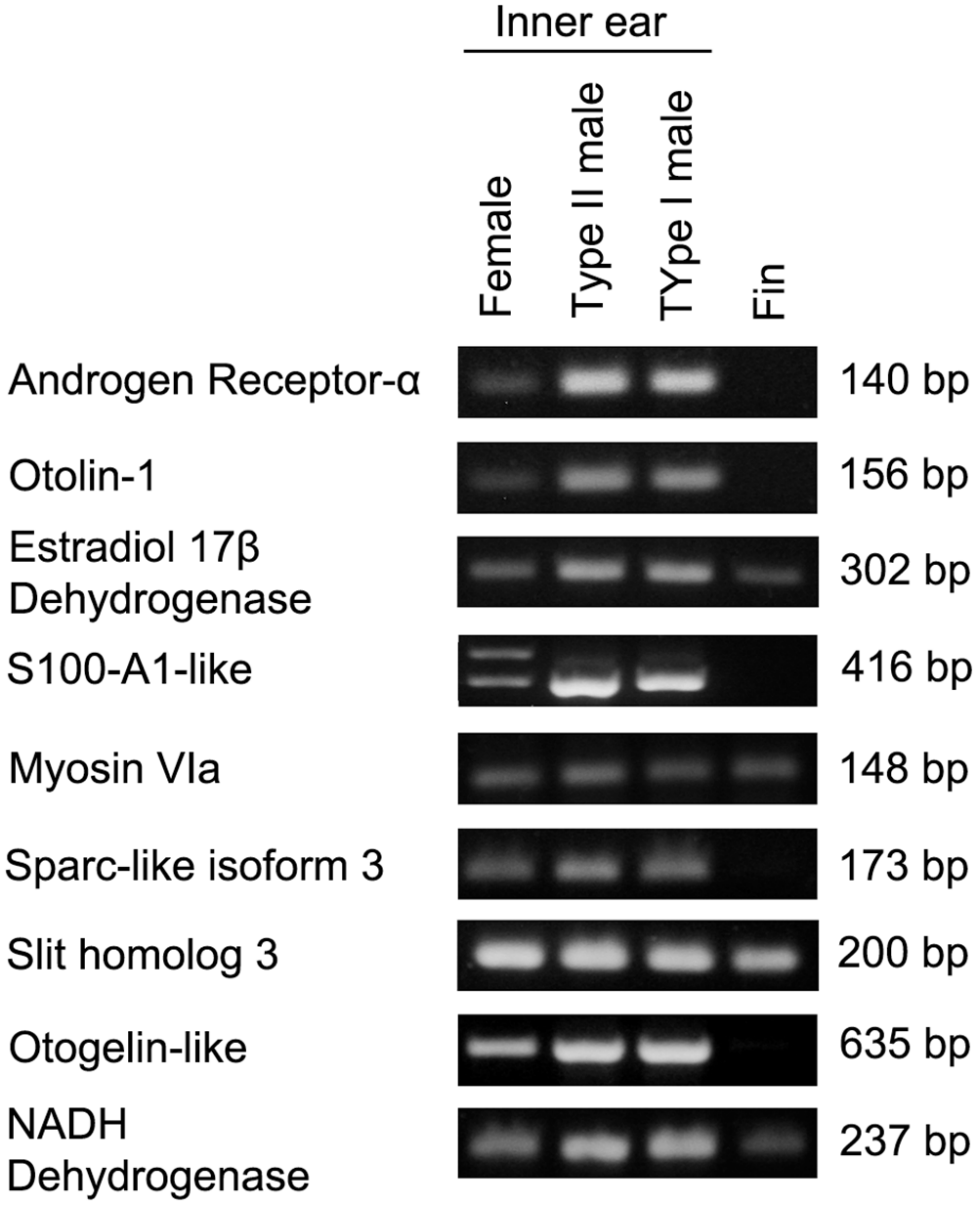
RT-PCR validation of midshipman inner ear gene expression. All nine genes (myosin VIA, estradiol 17β-dehydrogenase 12b, NADH dehydrogenase, S100-A1-like, SPARC isoform 3, otogelin-like, otolin-1, androgen receptor α, and slit homolog 3) were expressed in saccular tissue, while four genes were expressed in the fin. Band intensity is not a reflection of relative levels of gene expression.

To instill additional confidence in our RNA-Seq data, we validated gene expression by RT-PCR amplification of *myosin VIA*, *estradiol 17β-dehydrogenase 12b*, *NADH dehydrogenase*, *S100-A1-like*, *SPARC-like isoform 3*, *otogelin-like*, *otolin-1*, *androgen receptor α*, and *slit homolog 3*. We used saccular cDNA from all three sexual phenotypes while fin cDNA was used for qualitative comparison of inner ear gene expression. DNA sequencing confirmed the identity of all PCR products. Several of these genes (*otolin-1*, *S100*, *myosin VIa*, *otogelin*, *sparc*, and *slit*) are expressed in the inner ears of other vertebrates and may be important for inner ear development or maintenance [32–37]. *Estradiol 17β-dehydrogenase 12b*, which converts estrone into the more potent estradiol-17β, was chosen because estrogen plays a significant role in seasonal auditory plasticity in this species [9,38]. Similarly, androgen receptor-α plays a role in hormone signaling. Lastly, NADH dehydrogenase is a ubiquitous housekeeping enzyme that served as a positive control. As shown in Fig 5, all nine transcripts were expressed in the saccules of all three sexual phenotypes. We detected two S100-A1-like transcripts in the saccule of reproductive females, suggesting expression of multiple S100 isoforms in this reproductive phenotype. Estradiol 17β-dehydrogenase, myosin VIa, and slit homolog 3 were also expressed in fin, as is the housekeeping gene NADH dehydrogenase. It is important to note that band intensity is not a reflection of relative gene expression levels.

### Expression analysis

We used kallisto to analyze the relative expression levels (normalized by TPM) between the three summer (reproductive) sexual phenotypes and a newly generated dataset from the saccules of winter, non-reproductive females. In order to gain a better understanding of relative gene expression, we translated all 79,816 in ATs in 6 frames and considered the longest peptide in each case. The median size was 78 amino acids, which improved to 94 amino acids after considering only those 28,618 ATs having matches with *L*. *crocea* genes, consistent with the close concordance between the *P*. *notatus* and *L*. *crocea* genomes (Fig 4). Many of the longest translated peptides in our dataset had known inner ear-related functions (e.g., otogelin-like, collagen, otolin-1-a-like). Two ATs (combined_assembly_rep_c74637, combined_assembly_rep_c33111, see S2 Table) are noteworthy among the rest, because, in addition to long translation product, they were highly expressed in saccules from all three sexual phenotypes, but expressed at very low levels in the saccules of winter non-reproductive females. AT c74637 matches a receptor-type tyrosine-protein phosphatase in *L*. *crocea*, whereas AT c33111 matches the epiphycan (*epyc*, formerly called *DPSG3*) gene, a proteoglycan associated with the extracellular matrix [39].

We then reduced our AT dataset to 8,946 unique protein-coding genes by eliminating redundant gene IDs and used DESeq2 to analyze differential expression between sexual phenotypes using the full suite of expressed genes (S3 Table). We first compared saccular expression levels between reproductive and non-reproductive females, given that there is a known reproductive-state dependent difference in saccular sensitivity in this phenotype [8]. 769 transcripts were significantly differentially expressed between the female reproductive conditions, with 428 transcripts up-regulated in non-reproductive females and the remaining 341 up-regulated in reproductive female saccules (S4 Table). The most highly up-regulated genes in reproductive females were a transcriptional regulator, a protein of unknown function, an actin isoform, and neuroserpin. By contrast, non-reproductive female saccules showed substantial up-regulation of a nucleotide excision repair protein, a membrane protein related to vesicle trafficking, and a kinase with potential growth factor activity. Within the entire list of differentially expressed transcripts are several functional groupings, including Wnt and Notch signaling components, hormone receptors, and planar cell polarity proteins (S4 Table). Many of the up-regulated genes in the reproductive female saccules were also highly expressed in type I and type II males collected during the breeding season, suggesting that this set of genes is associated with the summer reproductive condition of the animals, or with the increased metabolic needs of more active animals.

We then compared relative gene expression between the two male phenotypes. Both were collected during the reproductive season from the same nests, so any differences may reflect true differences in auditory structure or function. Forty-seven genes were differentially expressed (p<0.05), with 16 of these genes more highly expressed in type II males and the remaining 31 genes up-regulated in type I males (Table 4). Of the 16 genes up-regulated in type II males, six have known roles in the inner ear, including two genes associated with deafness (polycystin/polycystic kidney disease 1, myosin XV), and a putative component of the mechanotransduction channel, transmembrane channel-like protein-2 (TMC2) [40–43]. It is important to note that none of the 47 differentially expressed genes was up- or down-regulated more than 2.3 fold, suggesting that saccular transcriptomes are generally similar between the two male sexual phenotypes.

**Table 4:** Protein-coding genes that are differentially expressed in type I vs. type II males. All genes shown here are significantly different at the p<0.05 level. *For this analysis we shown the raw p-values, as the adjusted p-values were all non-significant. Gene expression values are given in transcripts per million reads (TPM). ID numbers correspond to the gene table in S3 Table. Gene names in bold indicate genes with known inner ear function.

Gene ID	Type 1 Male		Type 2 Male	Log2 Fold Change	P-value*	Annotation
rna14389	1.145	79.624	2.233	0.027	FH1/FH2 domain-containing protein 3
rna9888	9.397	331.811	2.160	0.019	**transmembrane channel-like protein 2**
rna9921	5.228	216.148	2.079	0.025	**unconventional myosin-XV-like**
rna29562	0.348	27.709	2.061	0.046	SUMO-specific isopeptidase USPL1 isoform X1
rna18544	0.376	29.217	2.041	0.048	myosin light chain phosphorylatable fast skeletal muscle
rna8011	0.600	46.056	2.031	0.050	zinc finger FYVE domain-containing protein 1-like
rna4238	4.075	123.078	2.017	0.035	**protein piccolo**
rna28078	3.005	104.351	1.964	0.041	small nuclear ribonucleoprotein 70kDa (U1)
rna1625	4.266	113.672	1.942	0.040	GIPC PDZ domain containing family member 3
rna23069	2.748	63.058	1.924	0.042	DEAD (Asp-Glu-Ala-Asp) box helicase 56
rna11095	10.830	216.498	1.916	0.037	G patch domain containing 8
rna3394	10.917	238.319	1.826	0.045	SH3 and multiple ankyrin repeat domains 3
rna15732	9.395	228.236	1.806	0.045	**polycystic kidney disease 1**
rna14219	20.636	498.566	1.704	0.045	myelin protein P0
rna5121	59.738	1248.786	1.688	0.046	**parvalbumin thymic CPV3-like**
rna28310	33.979	579.490	1.680	0.049	**otoferlin-like**
rna16469	66.782	83.226	-1.674	0.049	tristetraprolin-like
rna2246	64.042	74.990	-1.715	0.044	DIP2 disco-interacting protein 2 homolog B (Drosophila) transcript variant X1
rna24775	190.240	137.788	-1.745	0.039	60S ribosomal protein L10 isoform X1
rna2503	152.668	156.818	-1.830	0.032	sodium- and chloride-dependent taurine transporter-like transcript variant X1
rna22205	35.062	24.016	-1.890	0.042	tumor necrosis factor alpha-induced protein 2-like
rna6299	20.502	13.983	-1.895	0.046	ATPase Na+/K+ transporting beta 4 polypeptide
rna27958	13.859	8.149	-1.955	0.041	tubulin beta-5 chain transcript variant X1
rna4791	27.517	30.607	-1.956	0.034	pre-B-cell leukemia homeobox 4
rna27139	36.959	25.238	-1.958	0.035	coronin-1C-like transcript variant X2
rna6436	44.531	47.917	-1.964	0.031	R3H domain containing 2 transcript variant X2
rna27282	29.525	9.920	-1.980	0.048	zinc transporter 8-like transcript variant X2
rna6012	63.527	22.503	-1.987	0.035	fibronectin-like
rna14834	13.430	6.153	-1.999	0.048	A disintegrin and metalloproteinase with thrombospondin motifs 2-like
rna15798	17.939	10.022	-2.023	0.035	glutathione reductase mitochondrial isoform X1
rna13147	6.220	1.809	-2.035	0.049	protein FAM46A-like
rna3689	24.213	11.572	-2.035	0.040	microtubule-associated protein RP/EB family member 2 transcript variant X1
rna4589	15.380	6.222	-2.042	0.043	zinc binding alcohol dehydrogenase domain containing 2
rna4812	53.421	36.259	-2.044	0.025	lipid phosphate phosphohydrolase 3-like
rna19828	59.271	32.913	-2.073	0.029	HECT and RLD domain containing E3 ubiquitin protein ligase 3
rna13994	7.565	2.360	-2.074	0.044	speckle-type POZ protein-like
rna2479	324.443	196.246	-2.129	0.016	exostosin-1b-like
rna20165	24.052	5.942	-2.138	0.034	collagen alpha-1(X) chain-like
rna3357	117.153	64.845	-2.175	0.017	armadillo repeat containing 10
rna7088	60.235	29.556	-2.180	0.019	ceramide synthase 2
rna23738	41.067	20.468	-2.189	0.023	adipocyte plasma membrane associated protein
rna9281	61.557	47.899	-2.190	0.014	creatine kinase U-type mitochondrial-like
rna4076	218.209	86.374	-2.216	0.014	RNA-binding motif protein X chromosome isoform X2
rna11578	11.819	2.980	-2.217	0.033	BMS1 ribosome biogenesis factor transcript variant X1
rna29385	22.783	3.247	-2.217	0.033	histone H2A
rna9391	65.336	83.874	-2.220	0.015	vacuolar protein sorting-associated protein 13D-like
rna13050	29.650	14.153	-2.288	0.020	muscleblind-like protein 3 transcript variant X2

Finally, we compared expression levels between reproductive females and type II males. Recent evidence suggests that type II males also demonstrate seasonal auditory plasticity, and type II males likely use acoustic cues for spawning nest identification to facilitate successful “sneak” spawning behavior [2,3,16, 44]. Sixty-one transcripts were differentially expressed between these two sexual phenotypes, with 41 up-regulated in type II males relative to reproductive females. Nine of the differentially expressed transcripts have known inner ear function (Table 5, bold text), with 8 of these 9 expressed significantly more in type II males than in females. The otolith matrix protein *otolin 1* is the only known inner ear gene significantly up-regulated in reproductive females (vs. type II males). Four inner ear transcripts that show reduced expression in reproductive females, *TMC2*, *myosin XV*, *otoferlin-like*, and *polycystin*, are significantly reduced in type I males as well (Table 4). *TMC2* is also highly expressed in saccules from non-reproductive females, while these other three inner ear genes are not upregulated in non-reproductive females (S3 Table). These analyses suggests that saccular gene expression is generally similar between the three reproductive morphs, but that expression in type I males and females is more similar to each other than either is to type II males.

**Table 5:** Protein-coding genes that are differentially expressed in reproductive females vs. type II males. All genes shown here are significantly different at the p<0.05 level. *For this analysis we shown the raw p-values, as the adjusted p-values were all non-significant. Gene expression values are given in transcripts per million reads (TPM). ID numbers correspond to the gene table in S3 Table. Gene names in bold indicate genes with known inner ear function.

Gene lD	Reproductive Female	Type 2 Male	Log2 Fold Change	P-value*	Annotation
rna10944	6.999	139.948	1.853	0.013	regulating synaptic membrane exocytosis 2 transcript variant X1
rna3322	27.907	441.680	1.851	0.011	dynein axonemal heavy chain 5
rna9370	9.908	169.458	1.832	0.013	proprotein convertase subtilisin/ kexin type 5-like
rna1388	7.940	160.772	1.804	0.016	signal transducer and activator of transcription 5B-like transcript variant X2
rna25728	4.979	119.483	1.772	0.019	ATP-sensitive inward rectifier potassium channel 10-like
rna10276	6.242	68.208	1.758	0.019	phosphorylase b kinase gamma catalytic chain liver/testis isoform
rna10609	2.045	59.915	1.754	0.021	SRY (sex determining region Y)-box 2
rna28400	7.742	138.417	1.744	0.019	olfactory receptor 10A7-like
rna10722	1.495	40.307	1.734	0.023	zinc finger protein 385B
rna21855	8.429	120.618	1.721	0.019	neurofascin
rna25126	35.224	414.760	1.696	0.018	**Phosphatidylinositol phosphatase PTPRQ**
rna13414	0.989	30.003	1.692	0.026	SH3-containing GRB2-like protein 3 interacting protein 1 isoform X7
rna18753	1.432	47.270	1.670	0.028	protocadherin gamma-A2-like
rna3394	16.714	238.319	1.637	0.024	SH3 and multiple ankyrin repeat domains 3
rna13480	7.693	134.264	1.635	0.025	fibrillin-1-like
rna18150	27.276	153.668	1.635	0.025	**plasma membrane calcium transporting ATPase 2**
rna24034	28.391	341.207	1.609	0.025	receptor-type tyrosine-protein phosphatase mu-like
rna18949	2.462	51.783	1.600	0.034	**mucolipin-3-like**
rna9888	35.130	331.811	1.598	0.025	**transmembrane channel-like protein 2**
rna28438	0.643	28.013	1.574	0.037	protocadherin beta-16-like
rna19896	4.597	79.379	1.574	0.038	WD repeat and SOCS box-containing protein 1
rna2012	1.007	26.367	1.571	0.039	RAC-gamma serine/threonine protein kinase transcript variant X1
rna9921	16.988	216.148	1.565	0.029	**unconventional myosin-XV-like**
rna24984	13.990	171.703	1.553	0.032	helicase with zinc finger 2 transcriptional coactivator
rna28080	0.762	23.741	1.544	0.042	protein phosphatase 1 regulatory subunit 35-like
rna6734	0.932	26.691	1.544	0.042	obscurin-like
rna10521	21.177	252.161	1.544	0.030	nuclear receptor corepressor 1
rna12342	1.720	40.837	1.531	0.042	transmembrane protease serine 7
rna6766	7.790	81.393	1.505	0.041	**excitatory amino acid transporter 1-like**
rna20117	3.923	55.313	1.504	0.047	lysosomal-associated transmembrane protein 4B
rna23323	2.128	24.415	1.504	0.048	adhesion regulating molecule 1 transcript variant X1
rna19304	1.902	58.753	1.503	0.048	RAB member RAS oncogene family like 6
rna29233	6.332	79.600	1.503	0.043	protocadherin gamma-A11-like
rna25715	34.562	400.827	1.502	0.034	uncharacterized LOC104936481
rna9185	21.235	234.031	1.495	0.035	NFX1-type zinc finger-containing protein 1 isoform X2
rna20098	2.102	29.267	1.495	0.049	39S ribosomal protein L11 mitochondrial
rna28310	58.145	579.490	1.495	0.033	**otoferlin-like**
rna12343	5.110	60.497	1.486	0.047	suppressor of tumorigenicity 14 protein homolog
rna15732	27.314	228.236	1.456	0.041	**polycystic kidney disease 1 (autosomal dominant)**
rna7083	39.680	466.971	1.417	0.041	histone-lysine N-methyltransferase SETDB1
rna9163	114.919	60.226	-1.406	0.046	LIM domain-binding protein 1 transcript variant X1
rna26281	50.451	26.610	-1.423	0.044	coagulation factor XIII A chain-like
rna13553	86.773	48.150	-1.431	0.046	transcriptional coactivator YAP1
rna3879	154.361	103.430	-1.461	0.037	annexin A1
rna17374	45.024	18.896	-1.489	0.044	major histocompatibility complex class I-related gene protein-like
rna27963	172.383	110.372	-1.508	0.032	chromosome unknown open reading frame human CXorf56
rna16918	42.974	24.232	-1.530	0.033	transmembrane protein 106B
rna3357	141.118	64.845	-1.536	0.032	armadillo repeat containing 10
rna2246	127.126	74.990	-1.554	0.027	DIP2 disco-interacting protein 2 homolog B (Drosophila) transcript variant X1
rna8815	22.827	2.961	-1.565	0.038	testis development related protein transcript variant X2
rna4791	51.598	30.607	-1.577	0.030	pre-B-cell leukemia homeobox 4
rna3473	26.927	7.808	-1.603	0.034	proline-rich protein 5-like
rna16889	37.485	8.602	-1.641	0.031	zinc finger protein Pegasus-like
rna17369	51.073	19.513	-1.649	0.027	NudC domain containing 1
rna8728	37.671	23.010	-1.681	0.021	MICAL-like 1 transcript variant X2
rna13147	11.641	1.809	-1.684	0.027	protein FAM46A-like
rna23738	53.717	20.468	-1.726	0.021	adipocyte plasma membrane associated protein
rna12095	147.167	52.728	-1.754	0.014	**otolin 1**
rna24066	12.684	1.761	-1.795	0.018	cilia and flagella associated protein 44
rna14162	66.264	33.872	-1.885	0.010	ER membrane protein complex subunit 2
rna27192	86.936	28.339	-1.934	0.010	RNA binding protein fox-1 homolog 2-like

## Discussion

We have successfully employed RNA-Seq technology to analyze the saccular transcriptome from the plainfin midshipman fish. The combined dataset represents over 79,000 total assembled transcripts representing almost 9,000 unique genes. Although our transcriptome coverage is relatively low for individual datasets, it is considerably higher when we consider coverage across all three summer sexual phenotypes, with >8 million sequences used to generate the combined assembly. The number of unique expressed genes from the combined assembly is close to that found in a recent cochlear transcriptome sequencing study [45], suggesting that our estimate may be close to the actual transcriptome size in the inner ear generally. Our dataset is likely missing some important genes that are expressed at low levels, *e*.*g*., transcription factors such as *atoh1* that are transiently expressed in developing hair cells [46,47]. Nevertheless, we have developed a valuable resource for a range of future midshipman inner ear studies, as well as comparative studies of the auditory periphery.

The generation of a *de novo* transcriptome for plainfin midshipman inner ear required the use of several bioinformatics tools in combination in order to lend confidence to our AT assignments. While BLASTX and BLASTN analyses identified many of the same genes within our dataset, the two analyses did not yield identical results. Each algorithm employs a different search strategy and offers unique advantages. BLASTX searches for amino-acid sequence homology for each possible open reading frame within a nucleotide sequence. This was particularly useful for annotating our dataset because few genetic resources exist for midshipman fish, and protein sequences are much more highly conserved across taxa than nucleotide sequences. BLASTN lends the advantage that some ATs may fall outside of the coding regions of genes (*e*.*g*., 5’ and 3’ untranslated regions or introns), and BLASTX would miss these homologies. Although our dataset was enriched for mRNAs, there were likely a number of non-coding RNAs in our dataset (rRNAs, snRNAs, lncRNAs) that BLASTX failed to annotate. The combination of both analyses helped us to validate the quality of our dataset and to annotate a higher proportion of ATs than with either analysis alone.

### Functional Annotation

We verified the presence of expected inner ear-related genes within our dataset, lending confidence that our false negative rate for the combined transcriptome assembly is relatively low. First, through BLASTX analysis, the present study identified ATs that fall into several functional classes, including over 100 genes that are known to play a role in the inner ear. This “inner ear” class represents transcription factors required for hair cell differentiation (*brn3c*), otolith-associated proteins (*otolin-1*, *SPARC3*), glycoproteins crucial for inner ear structural integrity (e,g. *otogelin*), and hair bundle genes such as *myosins VIA* and *VIIA*. *Myosin VIA*, *otolin-1*, *SPARC3*, and *otogelin* expression was also verified by RT-PCR. Microarray-based transcriptome profiling has identified many of these so-called deafness genes in the ears of other vertebrates, including rodents and the aquatic frog *Xenopus* [17,19,20].

Zebrafish mutagenesis studies show conserved function of many deafness genes across vertebrate taxa, suggesting that the inner ear ATs found in the present midshipman study are functionally important for hair cell development and/or maintenance [33,48,49]. Second, BLASTN analysis for a set of known deafness genes reconfirmed the presence of many of these same genes within our combined dataset. Deafness-associated genes that are not apparent in our dataset may have been missed due to the large phylogenetic distance between plainfin midshipman and the available sequences for BLASTN queries, as some of these deafness genes have only been sequenced in mammals. It is also likely that a subset of mammalian deafness genes are not expressed in the fish saccule. Genes associated with fluid homeostasis via expression in the stria vascularis may be specific to mammals, as fish ears employ an alterative strategy for ion exchange [50,51].

We also detected several transcripts previously identified in the midshipman inner ear, including BK channel subunits and androgen and estrogen receptors [52]. Androgen and estrogen receptors were previously detected in the midshipman saccule and may mediate hormonally-driven auditory plasticity in this species [9,53]. How steroid hormones induce seasonal changes in auditory function is unknown, but it may be due in part to an increase in supporting cell proliferation and hair cell addition during the pre-nesting season, when circulating hormone levels peak [54]. Recent work shows that reproductive female midshipman have increased hair cell density, decreased hair cell death, and more small, immature-appearing hair bundles than their non-reproductive counterparts, suggesting that the increase in hair cell addition may be initiated during the pre-nesting period [11]. Consistent with this hypothesis, the present study demonstrates saccular expression of several genes associated with cell death, survival, and proliferation.

### Phylogenetic Comparisons

Comparison of our long assembled transcripts (>1000 bp) to several known fish genomes revealed the greatest similarity with the *L*. *crocea* and *O*. *niloticus* genomes. According to a recent reconstruction of the teleost fish phylogeny, sciaenid fishes (drums and croakers, including *L*. *crocea*) are classified as Percomorpharia, a newly defined taxonomic group that also contains the O. Tetraodontiformes (pufferfishes, represented in the present analysis by *T*. *rubripes* and *T*. *nigroviridis*) and the traditional O. Perciformes (which includes *G*. *aculeatus*, the 3-spined stickleback). By contrast, cichlids (represented here by *O*. *niloticus*), cyprinodontiform (*X*. *naculatus*) and beloniform (*O*. *latipes*) fishes are part of a monophyletic group referred to as the Ovalentariae [31,55]. Midshipman are batrachoidid fishes, which are an apparent outgroup from the “apical phylogenetic bush” that comprises these other taxonomic groups. Therefore, our comparative analysis of the midshipman saccular transcriptome is inconsistent with recent updates to the bony fish phylogeny. *L*. *crocea*, *O*. *niloticus*, and *P*. *notatus* are all soniferous fishes where hearing is important for intraspecific communication [56,57], so it is possible that similarities between saccular transcripts represent conserved evolution for acoustic communication. Not surprisingly, we found that fewer of our long transcripts matched to the *D*. *rerio* (zebrafish) or *S*. *salar* (Atlantic salmon) genomes, consistent with the relatively basal positions of these fishes within teleost phylogeny [31,58].

### Relative Expression Analysis

Female midshipman fish exhibit seasonal auditory plasticity, with summer (reproductive) females having a saccular-specific increase in sensory receptor density coupled with increased auditory sensitivity [7,8,10]. We therefore examined the relative expression levels between winter and summer females as a first step in identifying the genetic correlates of this seasonal plasticity. Over 700 differentially expressed genes were identified in this comparison, with more transcripts significantly up-regulated in non-reproductive female saccules. Differentially expressed genes fall into several functional categories, but it is notable that several members of the Wnt and Notch signaling pathways were evident in this data set. Both pathways play critical roles in hair cell development and regeneration, including determining the balance between cell proliferation and hair cell fate specification [59–63]. Estrogen signaling influences expression of Wnt-mediated genes in both neurons and osteoclasts [64,65], and functional interactions between these pathways are reported *in vivo* [66]. Similarly, cross-talk occurs between estrogen and Notch signaling, with estrogen promoting Notch-dependent proliferation of mesenchymal stem cells [67,68]. Collectively, these data are consistent with the hypothesis that pre-nesting estrogen spikes modulate Wnt and Notch signaling, leading to increased cell proliferation and hair cell addition in the saccule of reproductive females.

Transcript expression was generally similar between the two male sexual phenotypes, and between type II males and reproductive females. Interestingly, type II males had greater expression of several known inner ear genes than either of the other reproductive phenotypes. Of particular interest is expression of *TMC2*, a putative component of the hair cell transduction channel, and *myosin XV*, a known deafness gene expressed in hair cell stereocilia [40,43]. Also of interest is the up-regulation of genes involved in synaptogenesis, including neurofascin and two protocadherins (β16 and γA-11). The significance of these findings is unclear, but it suggests that there may be more hair cells, and or more synaptic connections, in type II male saccules than in saccules from other sexual phenotypes. Recent data suggest that type II males also undergo a seasonal change in auditory sensitivity and are just as sensitive as females and type I males during the summer breeding season [44]. Overall, our analysis suggests that saccular gene expression is generally similar between the three reproductive phenotypes, but that expression in type I males and females is more similar to each other than either is to type II males. Our results also suggest that greater differences exist between non-reproductive and reproductive states, at least for females. These differences may reflect seasonal auditory changes, or may be a result of the different habitats where these fish were collected, as all reproductive fishes were obtained from shallow-water nests while winter females were collected via trawls in deeper water. Our data are consistent with a recent study that found significant up-regulation of metabolic transcripts, including protein synthesis genes, in the inner ears of type I male reproductive midshipman fish [69]. However, that study did not examine type II males, which occupy a unique transcriptional niche in our dataset. Future deep sequencing on the Illumina platform will allow us to better characterize relative expression levels between sexual phenotypes and reproductive-state dependent conditions and to assess animals housed in similar conditions.

### Availability of supporting data

Data is available at NCBI under the Bioproject accession PRJNA200442.

## Supporting Information

S1 Table Known human deafness genes and their presence in the midshipman saccular transcriptome. (XLSX)

S2 Table Kallisto analysis of relative expression levels for all assembled transcripts. (XLSX)

S3 Table Kallisto analysis of relative expression levels for all protein-coding genes. (XLSX)

S4 Table Genes highly differentially regulated in reproductive female saccules as compared to non-reproductive females, based on DESeq analysis. (XLSX)
